# Longitudinal evidence linking childhood energetics, maturation, skeletal muscle mass and adult human male sociosexuality

**DOI:** 10.1098/rsos.241713

**Published:** 2025-05-07

**Authors:** Lee T. Gettler, Stacy Rosenbaum, Sarah Hoegler Dennis, Sonny Agustin Bechayda, Christopher W. Kuzawa

**Affiliations:** ^1^ Department of Anthropology, University of Notre Dame, Notre Dame, IN, USA; ^2^ Department of Anthropology, University of Michigan, Ann Arbor, MI, USA; ^3^ Office of Population Studies Foundation; Department of Anthropology, Sociology, and History, University of San Carlos, Cebu, Central Visayas, Philippines; ^4^ Department of Anthropology, Northwestern University, Evanston, IL, USA

**Keywords:** mating strategies, condition-dependent traits, body size, age at sexual debut, puberty

## Abstract

Humans exhibit variation in their strategic expression of longer-term versus shorter-term mating strategies, including sociosexuality, which is defined as their interest and engagement in sexual activity outside of committed partnerships. There is substantial interest in the factors that drive variation in these strategies between individuals. Early life energetic conditions and psychosocial adversity may play key roles in shaping the expression of shorter-term mating strategies, particularly for males, given male–male mating competition. Drawing on a multi-decade study in the Philippines, we tested for links between males’ early life growth/maturation, adult skeletal muscle mass and childhood experiences of adversity with age at sexual debut (*n* = 965) and adult sociosexuality (*n* = 1594 obs.). Males who experienced more favourable childhood energetics had sex earlier and had more unrestricted sociosexuality, but these patterns were explained by males’ adult skeletal muscle mass. Males who were more maturationally advanced in adolescence also had younger ages at sexual debut and more unrestricted sociosexuality. We did not find evidence supporting the hypothesis that males exposed to early life adversity (family instability and sibling death) and favourable energetic conditions would show ‘faster’ life history strategies. Our findings point to the importance of developmental growth and maturation trajectories in energetically challenging ecologies to males’ later-life mating strategies.

## 1. Introduction

Humans have a life history strategy that commonly involves prolonged socially and sexually monogamous bonds between two individuals [1–4]. However, they also exhibit substantial variation in sociosexuality—i.e. individual differences in attitudes towards and willingness to engage in sexual activity outside of committed partnerships [1,5]. Evolutionarily, it is plausible that a stronger orientation towards uncommitted sex (i.e. unrestricted sociosexuality) would have conflicted with longer-term mating strategies—extended partnering between males and females with cooperative parenting effort—by shifting resources to shorter-term strategies that prioritized mating effort [1,5,6]. Consequently, to avoid the reproductive costs of partnership dissolution/conflict and help ensure cooperative care for our costly, slow-developing offspring, it is thought that restricted sociosexuality and longer-term mating strategies were often beneficial to fitness for many males and females [1–4,7].

Yet, human mating strategies are often mixed, and unrestricted sociosexuality and shorter-term mating strategies can also enhance fitness outcomes under certain conditions [1,8]. Males with characteristics that females prefer in short-term partners could have offspring with multiple females in parallel, potentially enhancing genetic fitness. Similarly, females can occasionally enhance the genetic quality of their offspring through ‘extra-pair’ mating outside of committed partnerships [1,8]. There are also ethnographic contexts in which multiple mating by females is viewed as typical and/or a pathway to increasing sources of male investment in females’ offspring [8–10]. Thus, there is substantial theoretical interest in the factors that drive variation in these strategies across and within societies [1,5,11]. This includes whether individuals tend to adopt longer versus shorter-term mating strategies at least in part based on cues received during development [12–14]. Early life environmental inputs could be key contributors to these later strategies because of their influence during critical/sensitive developmental periods for numerous biological systems and social competencies [15–17]. Developmental effects on mating strategies, including sociosexuality, are important because of their potential for lasting effects on psychosocial functioning, with impacts on outcomes spanning from relationship quality/stability to reproductive health [5,6,18].

In Euro-American societies, adverse early life family experiences and insecure attachment profiles often predict behaviours consistent with shorter-term mating strategies, including having more sexual partners and earlier ages at sexual debut [19–22]. Drawing on life history theory, psychosocial acceleration models posit that such results are consistent with the hypothesis that humans adaptively calibrate to a ‘faster’ life history strategy prioritizing earlier reproduction and shorter-term mating behaviours in response to adverse (i.e. harsh/unpredictable) developmental experiences [23–25]. Such models attempt to establish parallels between the role of extrinsic mortality risks in shaping life history strategies *across* species and the contributions of adverse early life experiences to life history variation *within* species. According to this ‘acceleration’ perspective, sociosexuality would likely also respond to early life conditions, reflecting a plastic psychological orientation that could integrate with behaviour and physiology along a fast versus slow phenotypic continuum [12,13,23,24]. However, the validity of this framing has come under increasing scrutiny [14,26–29], and the relevance of human sociosexuality to a fast versus slow continuum has also recently been questioned [14].

A key gap in this literature is that few prospective longitudinal studies have explicitly tested for links between early life experiences and adult sociosexuality [13,14]. This is theoretically important because sociosexuality comprehensively captures mating orientations by measuring psychological and behavioural inclinations for short-term, uncommitted sex versus long-term partnering and sex within those committed bonds [5,30]. Moreover, most research in this area is from energy-replete populations in industrialized/post-industrialized contexts [31]. This is notable because there are a number of pathways through which early life nutrition may play a role in the development of male sociosexuality and mating strategies.

For instance, more optimal early life energetic conditions lead to better childhood growth, contributing to larger adult body size and greater skeletal muscle mass [32,33]. For males, greater body size, muscularity and strength have been characterized as condition-dependent traits that can contribute to success in male–male competition, acquisition of status and attraction of mating partners. As such, these are examples of somatic states or cues by which human males may strategically calibrate their investment in shorter-term versus longer-term mating strategies across the life course [1,30,34]. Evidence from a range of settings provides initial support for this framing, though most existing research has notably relied on cross-sectional analyses of adults rather than developmental or life course data [31]. For example, in Euro-American contexts, more muscular and stronger adult males have earlier ages at first sex and more lifetime sexual partners than their peers with less muscularity and strength [31,34]. Research in multiple non-industrialized, smaller-scale societies has also shown that males with larger/stronger phenotypes have greater reproductive success [35–37]. Although less tested, some preliminary evidence similarly links such muscular somatic phenotypes to more unrestricted sociosexual attitudes [30,38].

Moreover, these associations between male somatic phenotypes and mating strategies raise the possibility that any contribution of harsh and unpredictable early life environments to the development of male sociosexuality may be contingent on childhood energetics. Specifically, beneficial early life nutrition may be critical to developing a phenotype that facilitates shorter-term mating strategies [23,31]. Thus, within an acceleration model perspective, the potential accelerating effects of early life psychosocial adversity towards a faster life history strategy for males may be dependent on pairing with resources that are sufficient to support healthy growth [23].

An additional, complementary pathway through which beneficial early life nutrition may shape development of sexual behaviour and adult sociosexuality is through earlier pubertal maturation. This is because nutrition may affect the timing of reproductive steroids’ ‘organizing effects’ during the pubertal transition and adolescence. Based on animal models, one hypothesis proposes that the brain is more susceptible to organizing effects of androgens when the pubertal transition occurs earlier [39]. A small number of human studies of sociosexuality align with this hypothesis. In one study, typically developing people of both sexes with earlier pubertal timing had more unrestricted sociosexuality, and for males who required hormone-replacement therapy to initiate puberty, those receiving the intervention at younger ages had more unrestricted sociosexuality as adults [40]. Another found a similar correlation between earlier puberty and unrestricted sociosexual attitudes in men but not in women [41].

Thus, for males, the puberty-accelerating effects of beneficial childhood nutrition could hasten the timing of this sensitivity window in the brain. Moreover, better early life nutrition could likewise contribute to greater androgen production as males mature, since indicators of favourable early life nutrition are linked to higher circulating testosterone in adult human males [42,43]. These potential neurobiological and hormonal effects therefore represent plausible pathways through which early life nutrition could coordinate psychobiological aspects of sociosexuality and shorter-term mating strategies alongside somatic, condition-dependent traits (i.e. body size and muscularity) [6,44].

Here, we used data from a multigenerational study in the Philippines to investigate whether physical traits (i.e. growth/body size; pubertal maturation), childhood experiences of family instability and environmental cues of local mortality during childhood predicted sociosexuality in adulthood. Because we are focused on the emergence of mating strategies across development, we also tested whether these same predictors were associated with males’ ages at sexual debut. Prior results from this cohort showed that family instability and adverse childhood experiences were associated with males’ ages at sexual debut [45,46]. We build from those results by considering physical traits as predictors of age at first sex, including child growth as a moderator of links between psychosocial cues and socio/sexual profiles (age at sexual debut; sociosexuality).

Specifically, we drew on data collected during childhood, adolescence and adulthood from a large sample of males enrolled in the Cebu Longitudinal Health and Nutrition Survey (CLHNS), which is an ongoing birth-cohort study that began in Metropolitan Cebu (Philippines) in 1983. We first tested whether favourable early life nutrition predicted earlier ages at sexual debut (*n* = 965) and more unrestricted adult sociosexuality (*n* = 877; 1594 obs.). We also assessed whether pubertal maturation and adult skeletal muscle (fat-free) mass helped to explain potential associations between favourable early life nutrition and those socio/sexual outcomes.

Subsequently, we explored whether favourable early life nutrition moderated associations between early life cues of mortality and familial instability in predicting males’ later socio/sexual outcomes. We specifically tested whether there would be a stronger association between those early life psychosocial experiences and socio/sexual outcomes when paired with favourable early life nutrition compared to those without the coupling of those early environmental exposures.

## 2. Methods

### 2.1. Study population

**Figure 1.**
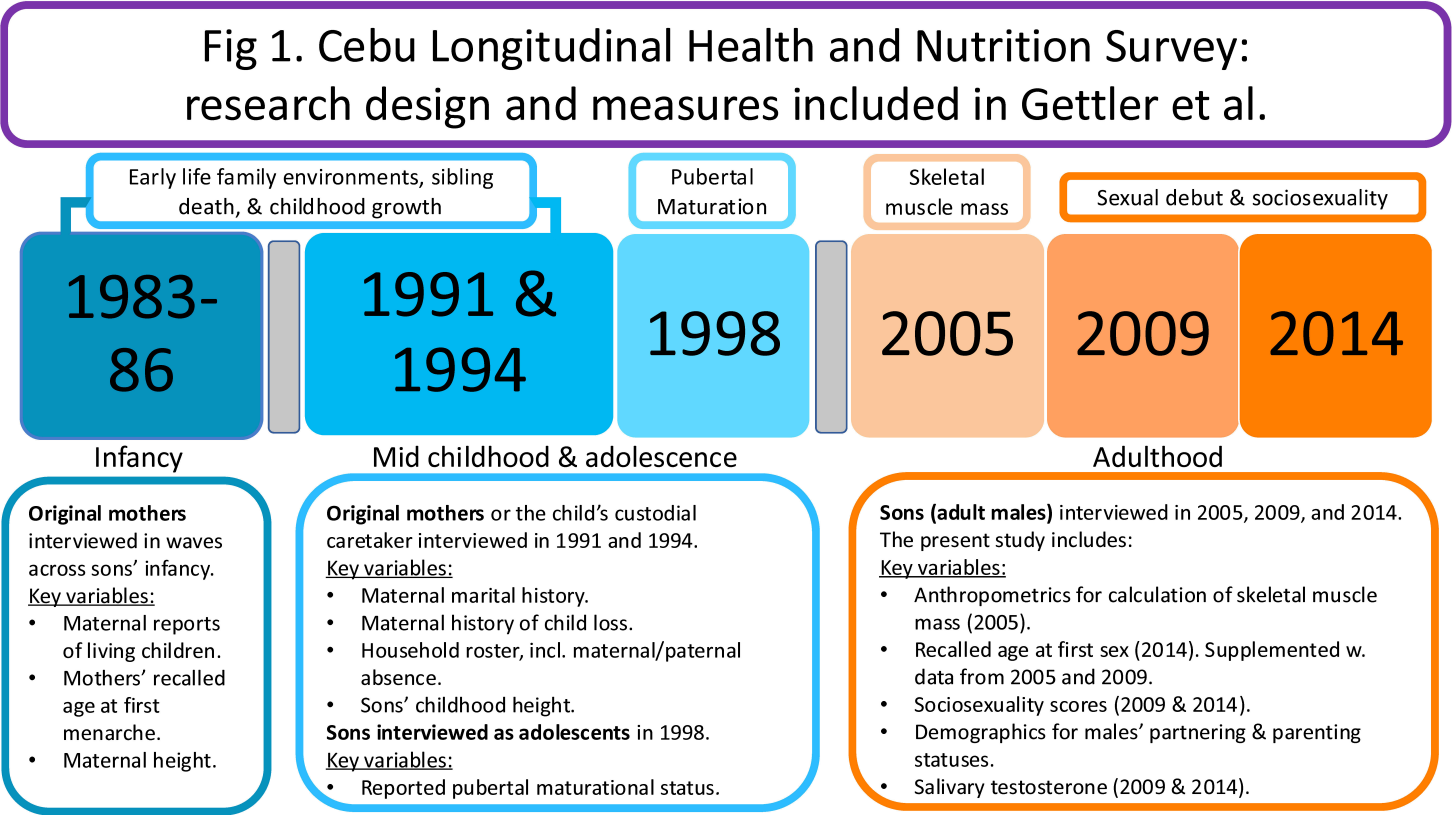
Visual overview of the longitudinal design for the study and the timing of data collection for key variables in the present analyses.

We drew on data collected between 1983 and 2014 as part of the CLHNS, a large, population-representative birth-cohort study of infants and their mothers in the Philippines [47]. In the present study, we drew on longitudinal data from families with infants identified as male at birth, who have been followed through multiple survey waves across their lives between 1983−1984 and 2014 (participants’ age in 2014: 30.5 ± 0.4 years). We used data collected from the original mothers in 1983−1986, 1991 and 1994, as well as their sons in 1998−1999, 2002, 2005, 2009 and 2014. In figure 1, we provide a visual overview of the longitudinal design for the study and the timing of data collection for key variables in the present analyses. During all surveys, socioeconomic, demographic and behavioural data were collected during in-home interviews administered by Cebuano-speaking interviewers. This research was conducted with informed consent and human subjects clearance from the institutional review boards of Northwestern University and the University of North Carolina. All participants provided written informed consent. We report descriptive statistics for the sample in table 1.

**Table 1.** Descriptive statistics.

	mean	s.d.	*N*
*physical characteristics*			
childhood height (cm)	132.23	6.93	965
advanced pubertal maturational status (% yes)^a^	41.7	—	973
FFM in young adulthood (kg)	46.90	5.81	973
*socio/sexual variables*			
sociosexuality (2009)	23.18	9.24	873
sociosexuality (2014)	20.35	8.81	721
age at first intercourse	18.55	3.20	940
*psychosocial environment variables*			
paternal instability (% yes)	18.6	—	973
maternal absence (% yes)	10.1	—	973
sibling death (% yes)	20.1	—	973
*other demographic variables*			
maternal education at birth (highest grade)^b^	7.42	3.76	966
birth order	3.31	2.30	973
participant education (highest grade)^b^	10.61	3.42	877

^a^ For maturational status, the percentage (% yes) refers to the % of males who were classified as being advanced for pubertal maturation when they were interviewed as adolescents in 1998−1999 (see §2).

^b^ Maternal and participant educational attainment are provided for descriptive purposes.

### 2.2. Key dependent variables

#### 2.2.1. Sociosexuality

In 2009 and 2014, participants filled out the revised version of the Sociosexuality Orientation Inventory (SOI-R) [5]. The SOI-R includes nine items, each of which is scored on a scale from 1 to 9. These nine items can be summed to form a measure of overall (or ‘global’) sociosexuality. They can also be broken into three sub-scales that measure psychometrically distinct components: sociosexual behaviour, desire and attitudes [5]. Among the study participants, the global score is robustly correlated to the sub-scales that it encompasses (2009, *r* = 0.69–0.83; 2014, *r* = 0.60–0.81) [6]. Because of these associations and to limit the number of analyses in the present study, we focus our main analyses on participants’ global sociosexuality scores, but we also report parallel analyses for the behaviour, desire and attitudes sub-scales in the electronic supplementary material. The reliability scores (Cronbach’s *α*) for the global sociosexuality scale were *α* = 0.71 (2009) and 0.68 (2014).

#### 2.2.2. Age at first sexual intercourse

Males reported their age at first sexual intercourse in 2014, and the vast majority (97%) had engaged in sexual intercourse by that time. For those who did not have 2014 data, we relied on comparable data from 2005 or 2009. For a small number of participants (2.7%), there were large discrepancies (>1 s.d.) between two waves for their reported age at sexual debut. For these participants, we triangulated across the three surveys to identify outlying values. We excluded two values +2.5 s.d. apart as missing.

### 2.3. Key independent variables

#### 2.3.1. Childhood and young adulthood anthropometrics

In Cebu, stunting was common in the 1980s, with the majority of rural (69%) and urban (62%) children in the study population showing stunting at 2 years old [48]. During the 1991 and 1994 surveys, anthropometric measurements were collected using standard techniques [49]. We calculated participants’ height for age (HFA) at each childhood time point as an indicator of their long-term nutritional experiences and energetic condition [50]. In Cebu, better childhood growth and nutrition are linked to advanced pubertal development during adolescence [45,48] and greater adult stature [32]. HFA in 1991 and 1994 were correlated at *r* = 0.79. We converted HFA in 1991 and 1994, respectively, to *z*-scores and averaged the values from the two time points. All participants had height measurements from both surveys except one.

In 2005, when the participants were young adults (age 21.5 ± 0.3 years), body weight (kg) and triceps, suprailiac and subscapular skinfold thicknesses (mm) were likewise measured using standard techniques [49]. Percentage of body fat was calculated from skinfold thicknesses using body density estimates and body composition predictive formulas from Durnin & Womersley [51]. We then calculated skeletal muscle (i.e. fat-free) mass (kg) based on percentage of body weight and converted the values to *z*-scores. A limited number of participants (4.5%) did not have anthropometric data in 2005 but did for a prior survey in 2002. We substituted their 2002 fat-free mass (FFM) data after conversion to *z*-scores. FFM in 2002 and 2005 were highly correlated (*r* = 0.82). We drew on this measure of FFM in young adulthood as a proxy for levels of skeletal muscle mass following reproductive maturation.

#### 2.3.2. Pubertal maturational tempo

In 1998−1999, adolescent participants reported pubic hair development by comparing themselves with drawings of pubic hair stages that were physician-validated among Filipino youth. Boys were considered more maturationally advanced if they classified themselves as being in the two most advanced pubic hair stages [43,45].

#### 2.3.4. Childhood experiences of unpredictability and cues of local mortality

Following prior Cebu analyses [45,48,52], we drew on childhood experiences of paternal instability and maternal absence as experiences of unpredictability as well as sibling death as an indicator of local mortality risks and harshness. We classified boys as having experienced paternal instability up to the 1994 survey (age 11.5 years (± 0.4)) if their father was deceased or absent, their mother was unmarried during their first year of life or beyond or their mother remarried during their childhood. Maternal absence was evaluated in 1991 and 1994. Sibling death was assessed via maternal reports of her children’s deaths after 1984 and up to 1994. These variables relied on maternal or caregiver interview data from surveys in 1983−1986, 1991 and 1994.

### 2.4. Covariates

#### 2.4.1. Sociodemographic covariates in adulthood

We included adult males’ partnering status and fathers’ co-residence with their biological children from the 2009 and 2014 survey waves. In the present analysis, we defined participants as partnered if they identified themselves as being legally married or cohabitating. In 2009 and 2014, fathers were defined as males who reported having one or more children. Among fathers in 2009 and 2014, respectively, 99% reported having at least one biological child. Fathers’ residence status was characterized by whether they resided with at least one of their biological children or none of them [53].

#### 2.4.2. Maternal covariates

Maternal height at the birth of their sons in 1983−1984 was measured using standard techniques [49], and mothers’ recalled age at first menarche was also recorded during that baseline survey. We included these maternal variables as covariates to partially adjust for inherited dimensions of height and maturational tempo [48]. We also included males’ birth order, which has been linked to the timing of sexual debut.

#### 2.4.3. Salivary testosterone

We collected saliva in 2009 and 2014 using similar morning–evening collection procedures, except that in 2014 we included repeated sampling for each subject. We adjusted the testosterone data for time of sample collection (see [54]). Testosterone concentrations were determined using an enzyme immunoassay protocol (Salimetrics; kit no. 1-2402). Inter-assay coefficients of variation for high and low kit-based control samples were as follows: 2009 (6.4 and 7.2%); 2014 (6.3 and 12.2%). We specifically included evening testosterone as a covariate in the present analyses based on past results linking evening testosterone and sociosexuality [6] and developmental experiences to adult testosterone [43,55].

#### 2.4.4. Infant growth

Earlier CLHNS research found that males’ growth (weight velocity) from birth to six months of age was linked to their age at sexual debut and their testosterone as young adults [43]. Adjusting our models for this infancy growth measure, as described in [43], did not meaningfully change our findings but did result in dropping over 40+ participants per model due to missing data. Thus, we did not include this variable in the models presented in the paper.

### 2.5. Statistical analyses

We conducted all statistical analyses using Stata v. 18.0 (Stata Corporation). We used survival time hazard regression models (Weibull distribution) to predict participants’ ages at first sex. We then used linear mixed models with maximum-likelihood estimation (Stata’s ‘mixed’ command) to predict participants’ sociosexuality scores. In each model for sociosexuality, we included a random intercept effect for each individual participant to account for the structure of the data, with individuals having two waves of sociosexuality observations included (2009 and 2014). We converted childhood HFA, adulthood FFM and other key continuous predictors to *z*-scores (s.d. units). We also converted males’ sociosexuality scores to *z*-scores.

For the analyses predicting each dependent variable, we first tested for bivariate relationships with the key energetic and somatic independent variables: childhood HFA, pubertal maturational status and young adulthood FFM. Next, we added maturational tempo together with HFA (model 1) and finally included young adulthood FFM (model 2). Finally, we added relevant covariates to the model, including maternal variables as well as adult sociodemographic and testosterone variables (sociosexuality analyses only; electronic supplementary material). For these analyses, we generated figures using predictive margins (adjusted predictions) in Stata.

For the moderation analyses focused on early life cues of mortality and familial instability, we included an interaction term between the respective environmental exposure (i.e. sibling death, maternal absence and paternal instability) and HFA (model 1), while adjusting for the other early life mortality/family exposures. We then added other relevant covariates to the model (model 2).

Following significant moderation analyses involving HFA as a continuous independent variable, we used the ‘margins, dydx’ command in Stata to conduct follow-up analyses to probe the interaction effects. For two-way interactions, this allowed us to directly test whether the association for HFA was significant for each sub-group in the dichotomous variable (e.g. experienced sibling death versus no sibling death). We evaluated statistical significance at *p* < 0.05.

## 3. Results

### 3.1. Key descriptive and bivariate statistics

We report descriptive statistics for the study sample in table 1 and illustrate key aspects of the longitudinal research design in figure 1. For males who had ever had sex by 2014, their median age at first sex was 18 years old and the interquartile range was 16−20 years old. Among those males, age at first sex was negatively correlated with sociosexuality in 2009 (*r* = −0.21) and 2014 (*r* = −0.20; both *p* < 0.001). Higher childhood HFA was associated with more advanced pubertal maturation status in adolescence (0.49 s.d. units; *p* < 0.001) and greater FFM in young adulthood (*r* = 0.60; *p* < 0.001). Advanced maturational status also predicted higher adult FFM (0.33 s.d. units; *p* < 0.001).

### 3.2. Physical development, sexual debut and sociosexuality

**Figure 2.**
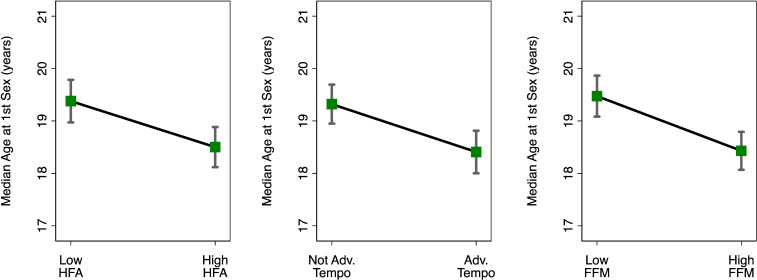
Males’ predicted median ages at sexual debut based on physical development and adult somatic characteristics. For visual purposes, we present predicted median ages based on males having relatively higher (+1 s.d. above the mean) or lower (−1 s.d. below the mean) scores for childhood HFA and FFM in adulthood (table 2). Adv, advanced; Mat, maturational status. Error bars represent 95% CI.

We first evaluated whether developmental characteristics (childhood HFA, maturational status) and adult FFM predicted males’ ages at first sex in bivariate analyses. In those analyses, greater childhood HFA, more advanced pubertal maturational status and higher FFM in young adulthood were all independently predictive of earlier sexual debut (all *p* < 0.001; table 2). Males’ predicted median age at sexual debut differed by 0.91 years based on advanced pubertal maturational status (figure 2). For visual purposes, figure 2 also shows predicted median ages for high versus low childhood HFA and high versus low FFM.

**Table 2.** Predicting males’ age at sexual debut and adult global sociosexuality from physical development and adult somatic characteristics. Males’ adult global sociosexuality scores were converted to *z*-scores. Reference groups for categorical variables: participants who were not categorized as advanced for pubertal maturation. Models that include advanced pubertal maturational status adjust for participants’ ages at the time of the adolescent interview (1998). Sociosexuality models also adjust for the year of data collection (i.e. 2009 or 2014). HR, hazard ratio.

	age at sexual debut (*n* = 965)								
	bivariate			model 1:			model 2:		
	HR	95% CI	*p*‐value	HR	95% CI	*p*‐value	HR	95% CI	*p*‐value
childhood HFA *z*-score	1.12	1.04, 1.19	0.001	1.08	1.01, 1.16	0.029	1.00	0.92, 1.09	0.941
advanced pubertal maturational status	1.26	1.11, 1.44	<0.001	1.20	1.05, 1.38	0.008	1.20	1.05, 1.38	0.009
adult FFM *z*-score	1.14	1.07, 1.21	<0.001				1.12	1.04, 1.21	0.002

	adult sociosexuality *z*-score (*n* = 877; obs. = 1594)								
	bivariate			model 1:			model 2:		
	coef	95% CI	*p*‐value	coef	95% CI	*p*‐value	coef	95% CI	*p*‐value
childhood HFA *z*-score	0.08	0.02, 0.14	0.014	0.06	0.001, 0.13	0.048	0.04	−0.04, 0.12	0.279
advanced pubertal maturational status	0.16	0.05, 0.28	0.006	0.13	0.01, 0.25	0.033	0.13	0.01, 0.25	0.032
adult FFM *z*-score	0.07	0.01, 0.13	0.023				0.03	−0.04, 0.11	0.403

Because childhood HFA predicts maturational status and adult FFM, the latter variables could help account for links between HFA and sexual debut. Thus, we then proceeded with testing the relative contributions of HFA and maturation in explaining age at sexual debut (model 1). When included together, both pubertal maturation and HFA remained significant predictors of age at sexual debut although the hazard ratios decreased slightly (both *p* < 0.05; table 2). This suggests that advanced pubertal status and better childhood growth each uniquely predicted age at sexual debut.

Finally, we added adult FFM as a predictor. In this model, more advanced pubertal maturation and higher FFM were predictive of earlier sexual debut (both *p* < 0.001), but childhood HFA was no longer significant (*p* > 0.9; table 2; model 2). This indicates that adult FFM helped to account for the association between better childhood growth and age at first sex. The results changed minimally with the addition of covariates (electronic supplementary material, table 1). In electronic supplementary material, figure 1, we also present Kaplan–Meier cumulative incidence plots corresponding to each of the core predictors in figure 2.

**Figure 3.**
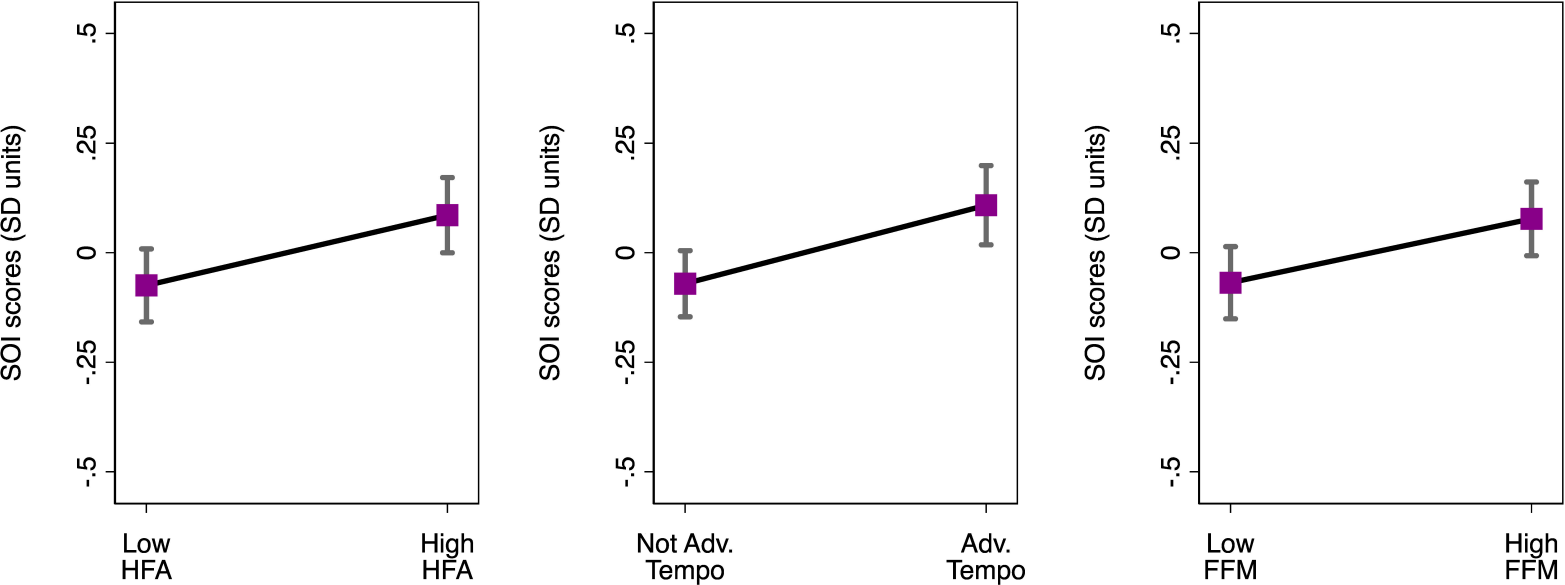
Males’ predicted global sociosexuality scores based on physical development and adult somatic characteristics. For visual purposes, we present predicted median ages based on males having relatively higher (+1 s.d. above the mean) or lower (−1 s.d. below the mean) scores for childhood HFA and FFM in adulthood (table 2). Adv, advanced; Mat, maturational status. Males’ global sociosexuality scores were converted to *z*-scores and are labelled in s.d. units. Error bars represent 95% CI.

Next, we tested a comparable progression of models predicting sociosexuality. In initial linear mixed models, males who were taller during childhood (HFA), had more advanced pubertal maturational status or had higher FFM in young adulthood, respectively, reported more unrestricted sociosexuality (all *p* < 0.01; table 2). The effect sizes (standardized *β*) for these findings were relatively small (0.07−0.16 s.d.; table 2 and figure 3). We then modelled HFA and pubertal maturation together, and the effect sizes for both predictors diminished modestly (both *p* < 0.05; model 1). In a subsequent model that added FFM in young adulthood, the association between advanced pubertal maturation and more unrestricted sociosexuality remained significant (model 2; *p* < 0.05). However, HFA and FFM in young adulthood were no longer significant predictors, suggesting shared variance (both *p* > 0.2; table 2; model 2). The inclusion of covariates did not meaningfully affect these results (electronic supplementary material, table 2). In particular, males with higher testosterone in adulthood reported more unrestricted sociosexuality (*p* < 0.05), but this did not affect the finding for maturational status (electronic supplementary material, table 2).

In the electronic supplementary material, we found complementary patterns for analyses focused on males’ sociosexuality sub-scale scores. Greater childhood HFA and more advanced pubertal status, respectively, were linked to higher sociosexual desire (both *p* < 0.05; electronic supplementary material, results). More advanced maturational status was also linked to higher sociosexual behaviour scores (*p* < 0.05). Young adulthood FFM was not significantly linked to the sub-scale scores, and the effect sizes were small (electronic supplementary material).

### 3.3. Childhood environments, sexual debut and sociosexuality

We then used moderation analyses to test whether beneficial childhood energetics (HFA) amplified associations between sibling death or family instability with age at sexual debut. We did not find statistically significant interactions between HFA and paternal instability or maternal absence, respectively, on age at first sex (both *p* > 0.7; electronic supplementary material, table 3).

**Figure 4.**
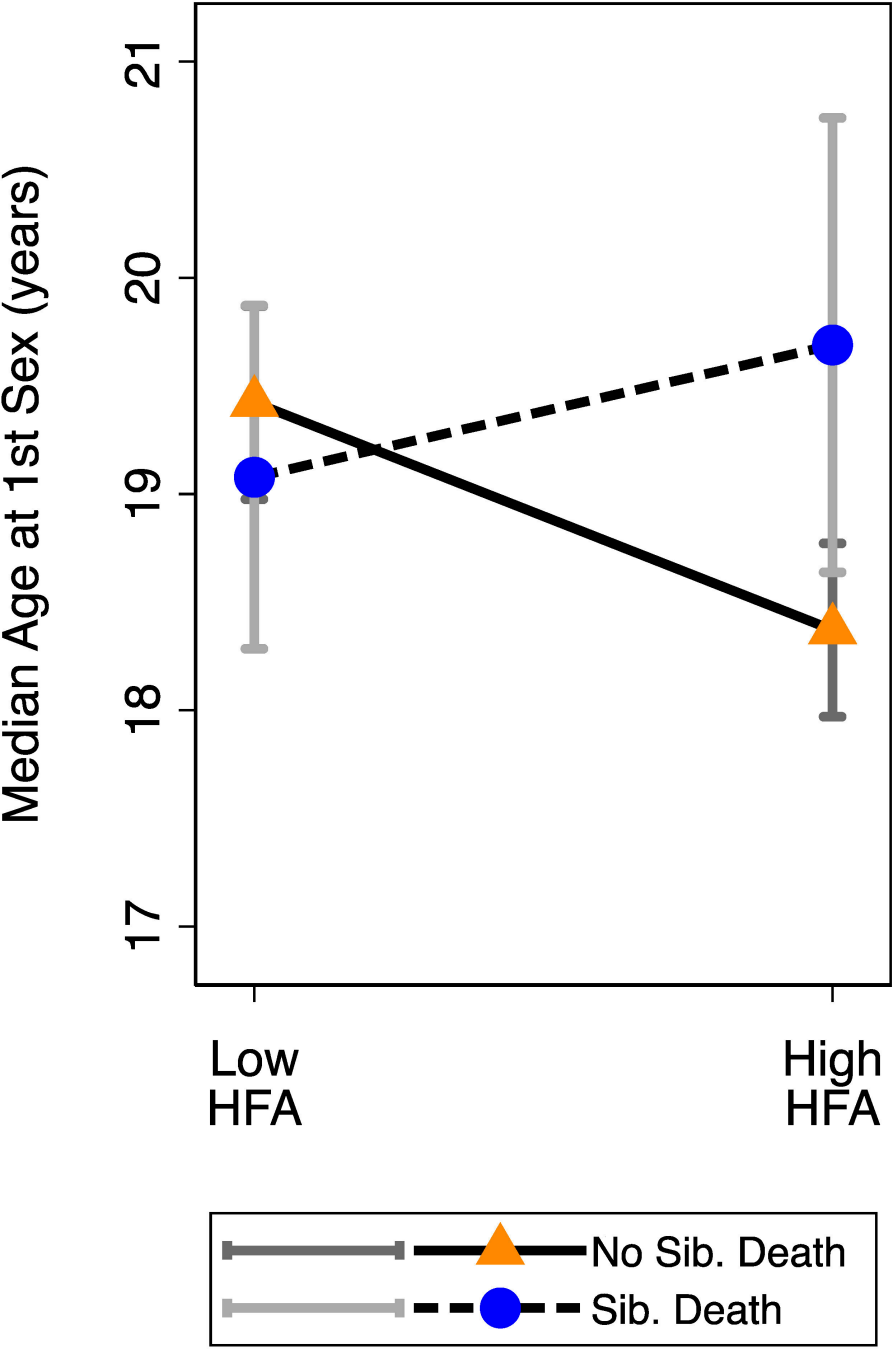
Males’ predicted median ages at sexual debut based on sibling death, moderated by childhood growth (HFA). For visual purposes, we present predicted median ages based on males having relatively higher (+1 s.d. above the mean) or lower (−1 s.d. below the mean) scores for childhood HFA (table 3). Error bars represent 95% CI.

We found a significant interaction between sibling death and HFA on age at sexual debut (*p* = 0.024; table 3). For males who did not experience the death of a sibling, those who had greater childhood HFA had significantly lower ages at first sexual intercourse compared to their peers with lower HFA (*p* < 0.001; figure 4). For those who experienced sibling death, childhood HFA did not significantly predict age at first sexual debut (*p* = 0.373). In figure 4, for visual purposes, we present predicted median ages at sexual debut based on high (+1 s.d. above the mean) and low (−1 s.d. below the mean) values for HFA and experiences of sibling death. After the addition of covariates, the significant interaction between sibling death and HFA changed minimally (electronic supplementary material, table 4), and there was a significant main effect for paternal instability, with those experiencing instability having earlier ages at first sex (*p* = 0.004).

**Table 3.** Predicting males’ age at sexual debut from (sibling death × childhood HFA; *n* = 965). Reference groups for categorical variables: participants who did not experience sibling death. These models also adjust for childhood maternal absence (*p* > 0.1) and paternal instability (*p* < 0.01) (see electronic supplementary material, table 4). Reference groups are coded 0, and, therefore, the given conditional effect (HR) represents the effect for that (reference) group: the effect for HFA is thus the effect of HFA on age at sexual debut for those who have not experienced a sibling death (adjusting for other covariates in the model). HR, hazard ratio.

	HR	95% CI	*p*‐value
childhood HFA *z*-score	1.14	1.06, 1.23	<0.001
experienced sibling death	0.88	0.74, 1.05	0.161
sibling death × childhood HFA	0.81	0.68, 0.97	0.024

Finally, we tested comparable interaction models predicting sociosexuality. There were no significant interactions between HFA and maternal absence (*p* = 0.762), paternal instability (*p* = 0.241) or sibling death (*p* = 0.882) on sociosexuality. Males who experienced sibling death reported more restricted sociosexuality than those who did not lose a sibling (*p* < 0.05). The main effects for paternal instability and maternal absence were not significant (all *p* > 0.4; electronic supplementary material, table 5).

## 4. Discussion

In this multi-decade longitudinal study, males who were taller for their age as children—an indicator of favourable early life energetic conditions in this population—had earlier ages of sexual debut and less restricted sociosexuality as adults. Those who were more maturationally advanced as adolescents or more muscular as young adults also reported younger ages of first intercourse and less restricted sociosexuality. We found that the links between childhood growth and males’ later socio/sexual profiles were explained by their muscularity in young adulthood, while adolescent maturational status predicted adult socio/sexual profiles independently of childhood energetics. In addition, our analyses did not support the hypothesis that early life psychosocial adversity would predict ‘faster’ life history strategies when coupled with favourable childhood nutrition. Collectively, these findings suggest the importance of early life energetic conditions and maturational timing for males’ sociosexuality and sexual behaviour, operating in part through the emergence of a somatic phenotype that has advantages for shorter-term mating strategies.

### 4.1. Physical development, sexual debut and sociosexuality

Our results are consistent with the hypothesis that early life energetic conditions affect aspects of male life history strategies by shaping their body size and composition and thereby their orientations towards shorter-term mating [1,30,34]. Using prospective data collected over multiple decades, we specifically showed that favourable early life energetic conditions were linked with younger ages at sexual debut and less restricted global sociosexuality, and these associations were largely accounted for by fat-free muscle mass in young adulthood. Advantageous developmental energetic conditions facilitate larger adult body size and greater skeletal muscle mass, and the link from childhood growth to adult FFM can be mechanistically understood by increasingly well-described biological processes [32,33,56]. This provides a rationale for why adult FFM helped to explain links between childhood growth and adult socio/sexual profiles when they were modelled together as predictors. While the sociosexuality effect sizes were comparatively small, the effect size in figure 3 reflects a 1.0 year difference in median age at sexual debut (0.33 s.d.) between males with relatively high versus low FFM in young adulthood. Although earlier ages at sexual debut may not translate to potential fitness implications in many contemporary human populations, earlier age of first reproduction has been linked to higher lifetime reproductive success in historical human populations [57], large-bodied non-human primates [58] and other large-bodied mammals [59,60]. While this is better studied in females because determining their age of first birth is more straightforward than conclusively determining age of first reproduction for males, similar benefits would likely also accrue to males [60].

The overall pattern of our findings aligns with Gangestad & Simpson’s [1] ‘Strategic Pluralism Theory’ of human mating strategies. This model suggests that humans have evolved to facultatively adjust their allocation of limited resources to shorter-term versus longer-term mating strategies in response to environmental cues. In some socio-ecological contexts, larger body size with greater FFM can enhance success in male–male competition, acquisition of social standing and attraction of mating partners [30,31,34]. In light of these advantages, socially mediated experiences (e.g. in competitive contexts) in adolescence can serve as external cues that shape strategic mating trajectories [1,61]. Recent research has also connected dimensions of personality, such as emotionality, to earlier ages at sexual debut and thereby more unrestricted sociosexuality [62]. These results point to individual differences that could moderate the effects of developmental energetic inputs on socio/sexual profiles. Given that better early life energetic conditions lead to greater childhood growth that ultimately contributes to larger adult body size and greater skeletal muscle mass [32,33], our findings specifically point to the importance of developmental growth experiences in energetically challenging ecologies in shaping these later-life facultative strategies through links to adult somatic phenotypes [32,33].

The present results also complement extensive past work showing that more muscular, stronger adult males report more success with shorter-term mating strategies (e.g. more lifetime sexual partners; earlier ages at first sex) [31], including unrestricted sociosexuality [38]. In a recent large meta-analysis, muscularity and strength were found to be the strongest physical trait predictors of mating attitudes/behaviours and reproductive success among human males [31]. These physical trait, fitness-related patterns have also specifically been documented in multiple non-industrialized societies [35–37]. Finally, our findings align with past longitudinal results from Cebu showing that a more rapid pace of infant weight gain in the ‘mini-puberty’ period, when testosterone is temporarily elevated in human male infants, predicted higher testosterone, FFM and engagement in shorter-term mating behaviours in adulthood [43]. These past ‘mini-puberty’ results hint at the potential for favourable early life nutrition to have organizing effects in the brain during sensitive developmental windows [43,55,63].

Testosterone is also an important mediator of male life history trade-offs between mating/competition and parenting effort, operating through somatic and activational psychobiological pathways in adulthood [64–67]. Past work in Cebu and other settings has shown that males with higher testosterone report more unrestricted sociosexuality, though with small effect sizes [6,44]. Thus, adult male testosterone is a plausible explanatory variable that could be linked to developmental energetic experiences, adult body composition and mating strategies. However, we did not find that the inclusion of testosterone as a predictor in our sociosexuality analyses meaningfully changed the effect sizes or significance of our key results.

We also found that males who were more maturationally advanced as adolescents had earlier ages at first sex and reported more unrestricted sociosexuality. These results were independent of childhood growth, despite favourable early life energetic conditions predicting more advanced pubertal maturation in Cebu [45]; the patterns were also not explained by adult FFM. The effect sizes for these results ranged from 0.16 s.d. for global sociosexuality to 0.29 s.d. for age at sexual debut. In complementary analyses, we also found that earlier maturers had higher sociosexual desire and behaviour scores, compared to later-maturing males, but the groups were similar for their sociosexual attitudes. These patterns suggest that earlier maturation could more strongly affect motivational (desire) aspects of sociosexuality and behavioural success in pursuing those desires, which are key factors in how individuals ultimately devote resources towards shorter- versus longer-term mating strategies. Meanwhile, males’ attitudes may be relatively more shaped by collective, broadly shared cultural norms and values (e.g. Catholicism and religiosity in Cebu) [5,68]. As we described earlier, a hypothesis based on non-human animal evidence posits that the brain is more susceptible to organizing effects of androgens when males experience the transition to puberty at younger ages [39]. Recent evidence from a study involving typically maturing individuals and those requiring hormonal intervention to initiate pubertal onset found support for this hypothesis for sociosexuality [40]. Our results can be viewed as consistent with this model.

However, additional bio-social perspectives may provide complementary insights into our findings. Meta-analyses of longitudinal studies indicate that advanced maturational status during adolescence predicts modestly earlier ages at first sex [69], aligning with our results. These associations are often viewed through bio-social perspectives suggesting that the hormonal shifts that occur during puberty contribute to secondary sexual characteristics, behavioural changes and shifts in social experiences, which are also responsive to puberty-driven physical changes (e.g. how peers respond to early maturers) [61,70]. For example, with the onset of increased testosterone production/activity, earlier-maturing adolescent males may be more driven to compete with other males for status and sexual/romantic opportunities than their later-maturing peers [65,71,72]. Early maturers also have physical characteristics (e.g. being comparatively taller and more muscular) that are often beneficial to competition and attractive to potential female partners [31], including in the Philippines [73]. This feedback-loop perspective is also potentially congruent with the Strategic Pluralism Theory of human mating [1,61].

### 4.2. Childhood environments, sexual debut and sociosexuality

It has been argued that early life psychosocial stressors might specifically lead to ‘faster’ life strategies when coupled with favourable energetic conditions [23]. Across three indicators of early life adversity, we did not find evidence supporting that prediction, and for one we observed results in the opposing direction. First, males who did not experience sibling death (i.e. less psychosocial adversity) had more unrestricted sociosexuality, on average, running counter to predictions of psychosocial acceleration models. Second, males who did not have a sibling die and experienced better childhood energetic conditions showed earlier ages of sexual debut, on average, than their peers. Males who lost a sibling and who also had advantageous early life energetic conditions did not exhibit younger ages at first sex (figure 4).

Relatively few prior studies have tested for direct indicators of extrinsic mortality rates and ages at sexual debut or first reproduction [74], especially in males; however, greater neighbourhood disadvantage, which can include violence exposure, has been linked to earlier ages at first sex and pregnancy/birth [75]. A study drawing on historical records in Canada and Europe found that individuals got married and became parents at younger ages if their parents had ever lost a child at any point, including before their birth [76]. Prior research in Cebu also showed that males who experienced sibling deaths became fathers at younger ages but did not have earlier ages at first sex [45]. If the present findings are replicated in other similar ecological settings, it could indicate that early life cues of harshness/mortality risk have the potential to dampen the otherwise accelerating effect of beneficial childhood energetic conditions on age at sexual debut.

In contrast, males who grew up with an unstable paternal presence had earlier ages at first sex, on average, after adjustment for covariates. This result replicates earlier results from Cebu for paternal instability and age at sexual debut, with an additional wave of longitudinal data and a 25% larger sample size [45]. This finding aligns with results from Euro-American contexts that have shown similar links between boys’ experiences of paternal absence/instability and earlier ages at first sex [77,78]. However, we did not observe complementary patterns for paternal instability predicting adult males’ sociosexuality. This contrasts with limited past longitudinal research that found that adults had more unrestricted sociosexuality if they experienced early life family instability [13].

There are a number of potential explanations for the divergent links between paternal instability with age at sexual debut versus sociosexuality in Cebu. First, the association with earlier ages at first sex could be driven by more proximate functional aspects of growing up in a household with an unstable paternal presence, such as less monitoring of behaviour and peer networks [77]. Such practical, monitoring dynamics in adolescence would not necessarily be expected to forecast sociosexuality measured over a decade later. An additional possibility involves a potential role for father-son relationship quality, particularly attachment. Attachment profiles, internal working models of social relationships and their development in the first years of life may be critical to the multi-faceted aspects of adult males’ sociosexuality, which encompasses desires and attitudes as well as behaviour regarding intimacy and partnering [79]. Those relationship effects may not be captured by our paternal instability variable, which is a coarse proxy and encompasses experiences up to ages 10−12 years old [13].

A final, related possibility is that age at sexual debut and sociosexuality do not constitute a cohesive, clustered set of traits along a fast-to-slow continuum that emerge in response to early life environmental adversity [14,26]. A number of critiques relevant to this point have been raised about the tenets of psychosocial acceleration models. Such critiques often question the validity of translating theoretical predictions that apply across species to within-species variation and plasticity. It is not clear whether correlated patterns of life history traits across species due to common selection pressures (e.g. extrinsic mortality) parallel within-species variation and intra-individual clustering of behavioural and cognitive traits in response to early environments [27–29]. Other questions have been raised about whether behaviours and psychological orientations typically associated with short-term mating strategies, including unrestricted sociosexuality, effectively map onto ‘faster’ life history traits, such as greater offspring production [80], in humans [14,26].

### 4.3. Limitations

Our study has limitations that merit discussion. Participants experienced family instability and sibling death at relatively low rates, and these proxy measures are somewhat coarse. In this setting, father absence or inconsistent presence across childhood could occur for multiple reasons, and not all of them have the same emotional and social valence. For instance, absence or instability could be due to marital separation or to fathers’ employment abroad [81]. Moreover, because the Cebu study was originally designed to focus primarily on mothers, the study’s fathering measures from the 1980s to 1990s are limited, including whether non-resident fathers remained involved with their children [55]. Such different sources of parental absence and varied levels of paternal involvement would likely have heterogeneous links with child outcomes, which could increase the possibility of type II errors. However, multiple studies from Cebu have demonstrated important effects of fathers’ co-residence and family roles to sons’ developmental trajectories [45,55,82], reflecting common Filipino cultural values for two-parent households and paternal contributions [83,84].

In our analyses, we also adjusted for maternal height and recalled age at menarche to help account for parent–child genetic confounding [85,86], though this approach is limited. The potential contributions of developmental environments to socio/sexual outcomes such as we focused on here can be overestimated if genetic factors are not taken into consideration [87,88]. For example, prior research has shown that genetic explanations help to account for links between father absence and age at sexual debut [87]. Pleiotropic genetic effects could also help explain associations between childhood growth and later socio/sexual profiles and merit consideration as potential contributors to the patterns we observed here.

## 5. Conclusion

In sum, in this multi-decade longitudinal study, we found results consistent with the idea that key developmental experiences—early life energetic conditions and maturational timing—may help shape the emergence of males’ sociosexuality and sexual behaviour. Early life growth may particularly help shape males’ socio/sexual profiles through contributions to a somatic phenotype that has advantages for shorter-term mating strategies.

## Data Availability

Data and relevant code for this research work are stored in GitHub: https://github.com/leegettler/RSOS_Cebu_April2025 and have been archived within the Zenodo repository [89]. Supplementary material is available online [90].
